# Expansion of Shiga Toxin–Producing *Escherichia coli* by Use of Bovine Antibiotic Growth Promoters 

**DOI:** 10.3201/eid2205.151584

**Published:** 2016-05

**Authors:** Jong-Chul Kim, Linda Chui, Yang Wang, Jianzhong Shen, Byeonghwa Jeon

**Affiliations:** University of Alberta, Edmonton, Alberta, Canada (J.-C. Kim, L. Chui, B. Jeon);; Beijing Key Laboratory of Detection Technology for Animal-Derived Food Safety at China Agricultural University, Beijing, China (Y. Wang, J. Shen)

**Keywords:** Shiga toxin–producing Escherichia coli, STEC, E. coli, bovine antibiotic growth promoters, Stx-encoding bacteriophages, bacteria, antimicrobial resistance

## Abstract

Antibiotics are routinely used in food-producing animals to promote growth and prevent infectious diseases. We investigated the effects of bovine antibiotic growth promoters (bAGPs) on the propagation and spread of Shiga toxin (Stx)–encoding phages in *Escherichia coli*. Co-culture of *E. coli* O157:H7 and other *E. coli* isolated from cattle in the presence of sublethal concentrations of bAGPs significantly increased the emergence of non-O157, Stx-producing *E. coli* by triggering the SOS response system in *E. coli* O157:H7. The most substantial mediation of Stx phage transmission was induced by oxytetracyline and chlortetracycline, which are commonly used in agriculture. bAGPs may therefore contribute to the expansion of pathogenic Stx-producing *E. coli*.

## Abstract 2

These growth promoters facilitate transfer of Shiga toxin–encoding phages in *E. coli*.

Antimicrobial agents are commonly used subtherapeutically as feed supplements to promote growth and to prevent infection in livestock. Despite growing public health concerns about resistance associated with agricultural use of antibiotics, their use in livestock production is anticipated to increase (*1*). Each year in the United States, 1,675 tons of nontherapeutic antibiotics are used in beef cattle, particularly in feedlots, which are intensive cattle-raising systems (*2*). According to a report from the US Department of Agriculture, ionophores, tylosin, chlortetracycline, and oxytetracycline are frequently given to feedlot cattle (*3*). Ionophores, such as monensin, are included in feed mainly to increase weight gain and to prevent bovine coccidiosis (*3*,*4*). Tylosin is used to prevent diseases (e.g., hepatic abscessation) and to promote growth in cattle (*3*,*5*), whereas chlortetracycline and oxytetracycline are used as feed supplements mainly to prevent bovine pneumonia and bacterial enteritis (*4*). Although antibiotics are usually added to feed, water, or both at subtherapeutic levels, at some feedlots, chlortetracycline and oxytetracycline are used at therapeutic levels to prevent infection, particularly when calves are first introduced into feedlots (*6*).

Shiga toxin–producing *Escherichia coli* (STEC), such as *E. coli* O157:H7, is the leading cause of hemorrhagic colitis and hemolytic uremic syndrome (*7*). Shiga toxin (Stx) is reportedly produced by ≈250 different O serotypes of *E. coli*; non-O157 STEC infection is becoming increasingly prevalent, accounting for up to 20%–50% of STEC infections in the United States (*8*). In particular, 6 serogroups (O26, O45, O103, O111, O121, and O145) are responsible for 83% of all non-O157 infections in the United States (*9*). The *stx* genes are encoded by lambdoid bacteriophages (*10*). Because secretion systems for Stx are lacking, the release of Stx is mediated through bacterial cell lysis by Stx phages in response to the induction of the SOS response (a cellular response to DNA damage) (*11*). Antimicrobial agents, particularly those that interfere with DNA synthesis (e.g., quinolones and trimethoprim), enhance the propagation of Stx phages and consequently increase Stx production (*12*). For this reason, antimicrobial drug treatment is not recommended for patients with enterohemorrhagic *E. coli* infection (*13*). In contrast, antibiotics are widely used as feed supplements in cattle, which are the primary natural reservoir for O157 and non-O157 STEC strains (*14*,*15*). *E. coli* is highly prevalent in cattle feces at levels ranging from 10^7^ to 10^9^ CFU/g (*16*), and *E. coli* O157 primarily colonizes the terminal rectum in cattle and is found in cattle feces at 10^3^–10^5^ CFU/g (*17*). Unlike humans, cattle are not susceptible to STEC infection because they lack Stx receptors (*18*); thus, antibiotics do not generate clinical problems in cattle. However, bovine antibiotic growth promoters (bAGPs) may induce the propagation of Stx phages and consequently facilitate the horizontal transfer of *stx* genes in *E. coli*. In this study, we investigated whether bAGPs can affect the propagation of Stx phages and contribute to the diversification of Stx-producing *E. coli*.

## Materials and Methods

### *E. coli* Strains, Plasmids, and Culture Conditions

We routinely maintained *E. coli* O157:H7 EDL933 and all *E. coli* isolates from cattle in Luria Bertani (LB) medium. The plasmid u66recA, a transcriptional fusion of *recA*::*egfp*, is described elsewhere (*19*). Detoxified EDL933 strains (Δ*stx*2::*Km* and *P_stx_*_2_::*gfp* in which *stx2* is replaced with a kanamycin resistance cassette and *gfp*, respectively) were constructed according to a method described by Datsenko and Wanner (*20*). The *stx*2 promoter region was PCR amplified from *E. coli* EDL933 with Pro_Stx2-F and Pro_Stx2-R primers (Table 1). The resulting 377-bp PCR product was purified, digested with *Xba*I, and ligated to an *Xba*I site located immediately upstream of the promoterless *gfp* gene in pFPV25.1 (*23*). *P_stx2_*::*gfp* was prepared by PCR with GFP_BGL_F and GFP_BGL_R (Table 1). The PCR product was cloned to a *Bgl*II site upstream of the flippase recognition target (FRT) in pKD13 (*20*). The FRT-flanked *P_stx_*_2_::*gfp* was amplified with PCR from pKD13 by use of pKD13-F and pKD13-R primers, and the amplicon was introduced to EDL933 harboring pKD46 by electroporation. The transcriptional *P_stx_*_2_::*gfp* fusion was constructed by replacing the *stx*2 gene with *gfp* in EDL933. The pKD46 plasmid was cured from the mutant by culturing at 37°C. A Δ*stx*2::*Km* mutant of *E. coli* O157:H7 EDL933 was constructed by replacing *stx*2 with a FRT-flanked kanamycin resistance cassette that had been PCR amplified from pKD13. The FRT-*Km*-FRT amplicon was introduced into EDL933 harboring pKD46 by electroporation (*20*). The allelic exchange was confirmed by PCR with the primer sets of Stx2-F and Stx2-R, Kt and K1. We added tetracycline (50 μg/mL), ampicillin (100 μg/mL), and kanamycin (50 μg/mL) to culture media when necessary.

**Table 1:** Primers used in study of expansion of Shiga toxin–producing *Escherichia coli* by bovine antibiotic growth promoters

Primer	Sequence, 5′ → 3′	Reference
Pro_Stx2-F	TAAGCATCTAGATTGCAGGATTAGTTACGT	This study
Pro_Stx2-R	TGCTTATCTAGAACAGGTGTTCCTTTTGGC	
GFP_BGL_F	TTCGAGCTCAGATCTCGGGGATCC	This study
GFP_BGL_R	TGCTTAAGATCTCGCATTAAAGCTTGCATG	
pkD13-F	CCAGGCTCGCTTTTGCGGGCCTTTTTTAT	This study
pkD13-R	GTGACACAGATTACACTTGTTACCCACAT	
Kt	CGGCCACAGTCGATGAATCC	(*20*)
K2	CGGTGCCCTGAATGAACTGC	
Stx2-F	GTCTGGTGCTGATTACTTCAGCCAA	This study
Stx2-R	ATTACACTTGTTACCCACATACCAC	
eaeA-F	GACCCGGCACAAGCATAAGC	(*21*)
eaeA-R	CCACCTGCAGCAACAAGAGG	
Ec1_uspA	CCGATACGCTGCCAATCAGT	(*22*)
Ec2_uspA	ACGCAGACCGTAAGGGCCAGAT	
O157F	CGGACATCCATGTGATATGG	(*21*)
O157R	TTGCCTATGTACAGCTAATCC	

### Antibiotics

Monensin, tylosin, chlortetracycline, oxytetracycline, neomycin, and sulfamethazine were purchased from Sigma-Aldrich (St. Louis, MO, USA). Ciprofloxacin was purchased from Enzo Life Sciences Inc. (Farmingdale, NY, USA).

### Measurement of *P_stx_*_2_*::gfp* Expression

Cultures carrying the *P_stx_*_2_::*gfp* promoter fusion were collected in the exponential phase by centrifugation (5 min at 6000 × *g*), and bacterial cells were resuspended in fresh LB medium to ≈4.0 × 10^7^ CFU/mL. After growing in LB broth supplemented with various concentrations of bAGPs and ciprofloxacin for 3 h, 200 µL samples were transferred to each well in a 96-well microplate and green fluorescent protein wavelength was measured with a fluorometer (FLUOstar Omega, BMG Labtech, Ortenberg, Germany). Fluorescence was monitored at excitation and emission wavelengths of 520 nm and 480 nm, respectively. Fluorescence intensities are reported in the instrument’s relative fluorescence units. The expression level of *rec*A was measured in the same way with *u66rec*A. The experiments were performed with triplicate samples and repeated at least 3 times.

### Stx Phage Induction

Stx phages were induced by monensin, tylosin, chlortetracycline, oxytetracycline, and ciprofloxacin. Because ciprofloxacin is a well-known Stx phage inducer (*12*), we used ciprofloxacin as a control. Lysogenic strains were grown in LB broth overnight at 37°C with shaking and diluted to an optical density at 600 nm of 0.1 in NZCYM broth (Amresco, Solon, OH, USA). The cultures were incubated at 37°C with shaking (200 rpm) for 18 h in the presence (0.01, 0.1, and 1 μg/mL) and absence (control) of the 5 antibiotics. After centrifugation at 5,000 × *g*, the supernatant was sterilized with a 0.22-μm filter and used immediately. We then added 10-fold serial dilutions of phage lysates to 1 mL of *E. coli* C600 at the stationary phase. Then, 3 mL of top agar supplemented with 5 mmol/L calcium chloride was added to this culture and the mixture was poured on an LB agar plate. The plates were incubated at 37°C overnight, and PFU were counted the next day.

### Stx Phage Transfer Assay

We investigated the transfer of Stx phages in the presence of bAGPs at different concentrations. Briefly, 5 mL of *E. coli* EDL933 Δ*stx*2::*Km* (donor) and 6 *stx*2-negative bovine *E. coli* strains (recipients) with ampicillin or tetracycline resistance were grown in LB broth at 37°C overnight. Overnight cultures were diluted 100-fold in fresh NZCYM broth and cultured until the early exponential phase for 3 h. The donor strain (≈10^4^ CFU/mL) was mixed with recipient strains (≈10^7^ CFU/mL) in the presence of different concentrations of bAGPs. The mixed cultures were incubated at 37°C overnight without shaking. After incubation, 100 µL of culture was spread onto sorbitol-MacConkey agar plates supplemented with kanamycin and ampicillin or tetracycline and incubated at 37°C overnight. We calculated the transduction frequencies by dividing the number of transductants by the number of recipients.

### Characterization of Transductants

Pink colonies growing on sorbitol-MacConkey agar supplemented with either kanamycin and tetracycline or kanamycin and ampicillin were regarded as presumptive transductants (i.e., recipients of the *stx*2-encoding phage 933W). The presumptive transductants of the *stx*2-encoding phage 933W were verified by performing multiplex PCR. PCRs were performed with specific primer pairs: Stx2-F and Stx2-R for the *stx*2 gene in 933W, eaeA-F and eaeA-R for *eae*A encoding intimin (*21*), Ec1-uspA and Ec2-uspA for the *usp*A gene encoding the universal stress protein in *E. coli* (*22*), and O157F and O157R for a region in *rfb*E (O-antigen-encoding) for the O157 serotype (*21*). Serologic tests were performed with O157 and O26 antiserum (Korea National Institute of Health, Osong, South Korea).

## Results

### Enhanced Propagation of Stx Phages in *E. coli* O157:H7 by bAGPs

**Figure 1:**
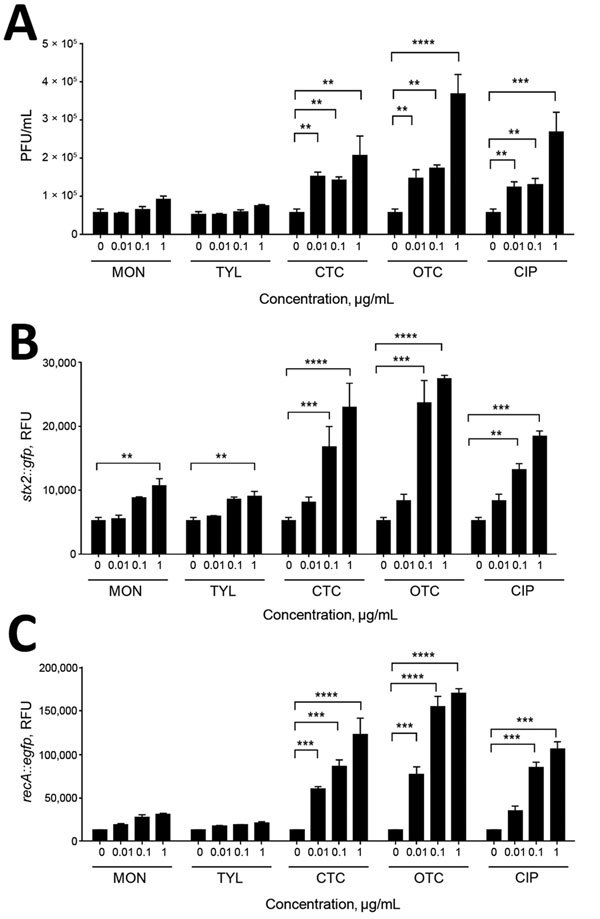
Induction of Shiga toxin (Stx)2 phage propagation and SOS response by bovine antibiotic growth promoters (bAGPs). A) Stx2 phage induction in *Escherichia coli* O157:H7 EDL933 after 3 h exposure to subtherapeutic concentrations of bAGPs, including monensin (MON), tylosin (TYL), chlortetracycline (CTC), and oxytetracycline (OTC). Ciprofloxacin (CIP) was a control for phage induction. *E. coli* C600 was used as a phage-susceptible strain. B) Induction of *stx*2 expression by bAGPs. Fluorescence from an *stx*2::*gfp* transcriptional fusion indicates the level of Stx2 phage induction. C) Induction of *recA* transcription by bAGPs. The level of *recA* expression indicates the level of SOS response induction. The results show means and SDs of 3 independent experiments. Statistical significance was analyzed by using the Student *t*-test. **p<0.01, ***p<0.001, ****p<0.0001.

To investigate the effect of subtherapeutic concentrations of bAGPs on the propagation of Stx phages, we first measured the level of Stx phage propagation after exposure of *E. coli* O157:H7 EDL933 to sublethal concentrations (1, 0.1, and 0.01 μg/mL) of common bAGPs, including monensin, tylosin, chlortetracycline, and oxytetracycline. Because the bAGP concentrations used in the study were markedly less than the MICs (Table 2), bAGP treatment did not affect the growth of *E. coli* O157 (data not shown). The propagation of Stx phages was induced significantly by chlortetracycline and oxytetracycline at concentrations as low as 0.01 μg/mL (Figure 1, panel A). Because *E. coli* O157:H7 EDL933 harbors 2 Stx prophages, BP-933W (*stx*2) and CP-933V (*stx*1) (*24*), the level of *stx*2 expression was specifically measured with an *stx*2::*gfp* fusion construct to determine the propagation level of Stx2 phage. Consistently, bAGP treatment substantially increased the level of *stx*2 expression (Figure 1, panel B). Because the primary mechanism for antibiotic-mediated induction of phage propagation is the SOS response, we also determined the level of *rec*A expression after exposure to bAGPs. Consistent with the changes in the level of Stx phage propagation (Figure 1, panels A, B), chlortetracycline and oxytetracycline significantly induced *recA* expression (Figure 1, panel C). Of note, chlortetracycline and oxytetracycline induced Stx phage propagation and *stx*2 expression at levels similar to those of ciprofloxacin, a DNA-damaging antibiotic frequently used as a phage inducer (Figure 1).

**Table 2:** MICs and MBCs of bAGPs for *Escherichia coli* O157:H7 EDL 933

bAGP*	MIC, μg/mL	MBC, μg/mL
Monensin	>1,024	>1,024
Tylosin	>1,024	>1,024
Chlortetracycline	4	8
Oxytetracycline	8	16

*bAGP, bovine antibiotic growth promoter; MBC, minimum bactericidal concentration.

### Increased Propagation of Stx Phages in Bovine STEC Strains by bAGPs

**Figure 2:**
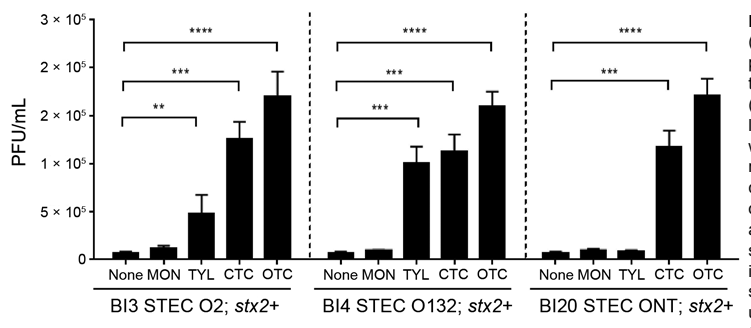
Induction of Shiga toxin (Stx)2 by bovine antibiotic growth promoters (bAGPs) in Shiga toxin–producing *Escherichia coli* (STEC) strains from cattle. The levels of Stx2 phage induction were examined with 0.1 µg/mL monensin (MON), tylosin (TYL), chlortetracycline (CTC), and oxytetracycline (OTC). ONT, O antigen nontypable. The results show means and SDs of 3 independent experiments. Statistical significance was analyzed by using the Student *t*-test. **p<0.01, ***p<0.001, ****p<0.0001.

When we further examined Stx phage induction by bAGPs with 3 *stx*2+/*stx*1– *E. coli* strains from cattle, we found that exposure to a sublethal concentration (0.1 μg/mL) of chlortetracycline and oxytetracycline significantly induced the propagation of Stx2 phage in the bovine STEC isolates, whereas 0.1 µg/mL of monensin did not induce phage propagation, and 0.1 μg/mL of tylosin exhibited strain-dependent variations in the phage induction (Figure 2).

### Transfer of Stx Phages in Bovine *E. coli* Isolates by Sublethal Concentrations of bAGPs

**Figure 3:**
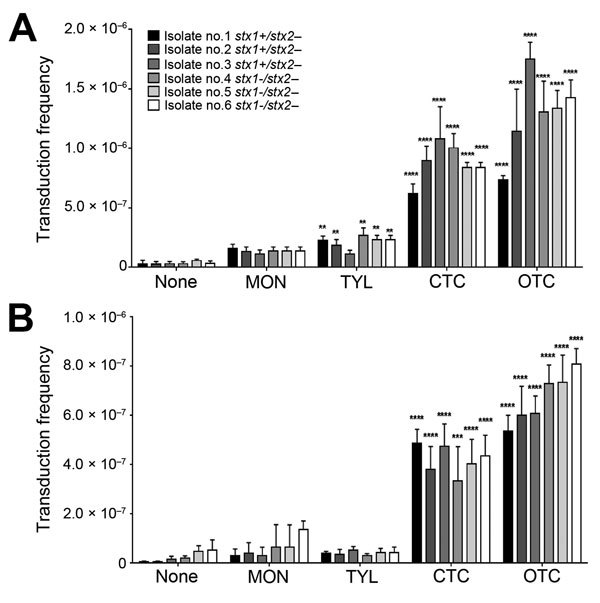
Emergence of Shiga toxin (Stx)2–positive strains of *Escherichia coli* from cattle (isolates 1–6) by subtherapeutic bovine antibiotic growth promoters (bAGPs) at 0.1 µg/mL (A) and 0.01 µg/mL (B). Frequency of transfer of Stx phages to bovine *E. coli* isolates. The Stx pages originated from a detoxified derivative of *E. coli* O157:H7 EDL933, where *stx2* was replaced with a kanamycin resistance cassette. The donor and recipient *E. coli* strains were co-cultured with or without monensin (MON), tylosin (TYL), chlortetracycline (CTC), and oxytetracycline (OTC). The transduction frequencies were calculated by dividing the number of transductants by the number of recipients. The results show means and SDs of 3 independent experiments. Statistical significance was analyzed by using the Student *t*-test in comparison with antibiotic-free cultures. **p<0.01, ***p<0.001, ****p<0.0001.

We determined the frequency of Stx2 phage transfer with 6 *stx*2-negative *E. coli* isolates from cattle, including 3 *stx*1+/*stx*2– *E. coli* strains and 3 *stx*1–/*stx*2– *E. coli* strains, by using a detoxified EDL933 derivative in which *stx*2 was replaced with a kanamycin resistance cassette. The donor *E. coli* (EDL933 Δ*stx*2::*Km*, a detoxified strain) and the recipient *stx2*-negative *E. coli* strains were co-cultivated in the presence of sublethal concentrations (0.01 μg/mL and 0.1 μg/mL) of bAGPs. The recipient bovine *stx*2-negative *E. coli* isolates are all sensitive to kanamycin and resistant to β-lactams or tetracycline, and the detoxified EDL933 derivative is resistant to kanamycin and sensitive to β-lactams and tetracycline. Therefore, the Stx phage transfer made the recipient strains resistant to both kanamycin and β-lactams or tetracycline. Subtherapeutic treatment of bAGPs, particularly chlortetracycline and oxytetracycline, substantially enhanced the transfer of Stx2 phage in *E. coli* (Figure 3, panel A). The transduction rate was slightly increased by 0.1 µg/mL tylosin but not notably affected by 0.1 µg/mL monensin (Figure 3, panel A). When the concentration of bAGPs was reduced to 0.01 μg/mL, tylosin did not mediate the Stx phage transfer. However, chlortetracycline and oxytetracycline significantly mediated the transfer of Stx phage in *E. coli* even at 0.01 μg/mL (Figure 3, panel B).

### Induction of Stx Phage Propagation by Therapeutic Concentrations of Chlortetracycline and Oxytetracycline

**Figure 4:**
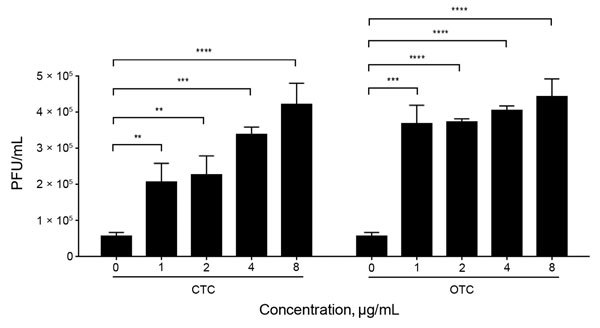
Induction of Stx2 phages by treatment with high concentrations of chlortetracycline (CTC) and oxytetracycline (OTC). The phage titer was determined in *Escherichia coli* O157:H7 EDL933 by treatment with 1 to 8 µg/mL of CTC and OTC. The results show means and SDs of 3 independent experiments. Statistical significance was analyzed by using the Student *t*-test in comparison with antibiotic-free cultures. **p<0.01, ***p<0.001, ****p<0.0001.

Whereas tylosin and monensin are used at low concentrations in cattle feed, chlortetracycline and oxytetracycline are sometimes used at therapeutic levels to prevent infection (*6*). Thus, we investigated the effects of high concentrations of chlortetracycline and oxytetracycline on the propagation of Stx phages. High concentrations of chlortetracycline significantly increased the propagation of Stx phages in a concentration-dependent manner, whereas the level of Stx phage induction by oxytetracycline is already significantly high at 1 μg/mL in comparison with higher concentrations of oxytetracycline (2–8 μg/mL) and even the highest concentration of chlortetracycline used in the study (8 μg/mL) (Figure 4). These findings demonstrate that therapeutic application of chlortetracycline may enhance the dissemination of Stx phages more significantly than subtherapeutic doses and that oxytetracycline is a highly potent inducer of Stx phage propagation even at low concentrations.

### Confirmation of Stx Phage Transfer by bAGPs

**Figure 5:**
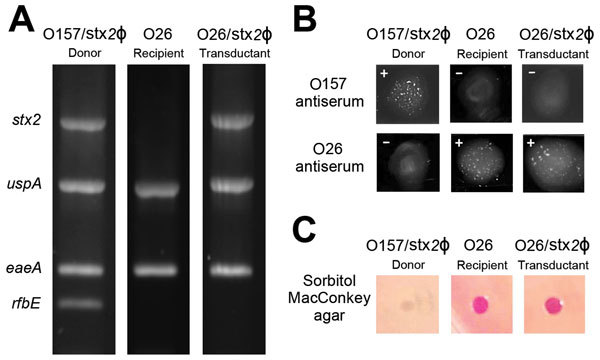
Transfer of Shiga toxin (Stx) phages by bovine antibiotic growth promoters (bAGPs) in *Escherichia coli* isolates from cattle**.** For the confirmation of Stx phage (Stx2Ф) transfer, *E. coli* O26 (*stx2*-negative, bovine isolate no. 1 in Figure 3) was used. A) PCR confirmation of the presence or absence of *stx*2, *usp*A, *eae*A, and *rfb*E_O157_ in EDL933 (Stx2Ф donor), O26 before transduction (Stx2Ф recipient), and O26 after transduction (transductant). B) Serotyping of *E. coli* O157 and O26. +, positive reaction; –, negative reaction. C) Sorbitol fermentation of donor, recipient, and transduced strains on sorbitol MacConkey agar plates. *E. coli* O157 does not ferment sorbitol, whereas non-O157 *E. coli* ferments sorbitol and produces pink colonies.

To confirm Stx phage transfer by bAGPs, we randomly chose transductant colonies from the co-culture experiment described earlier (Figure 3) for further verification with PCR to detect genes specific for the Stx2 phage (*stx*2), *E. coli* (*usp*A), *E. coli* virulence (*eae*A), and O157 serotype (*rfb*E_O157_) (Figure 5, panel A). Figure 5 shows representative data to exhibit the dissemination of the *stx2* gene to *stx*2-negative *E. coli* O26 (bovine isolate no. 1 in Figure 3) by exposure to bAGPs. We selected *E. coli* O26 because this serotype is the most frequently isolated non-O157 STEC (*9*). Treatment with bAGP changed bovine *E. coli* O26 from *stx*2-negative to *stx*2-positive. We also performed a latex agglutination test to confirm the serotype after transduction (Figure 5, panel B). The capability of sorbitol fermentation in non-O157 strains was confirmed by growing on sorbitol MacConkey agar plates (Figure 5, panel C). The results clearly showed that non-O157 *E. coli* horizontally acquired *stx*2 by phage transduction after exposure to bAGPs.

## Discussion

In livestock production, antibiotics are routinely added to feed for growth promotion and disease prevention. Although these AGPs are used at subtherapeutic concentrations, a substantial number of studies have shown that AGPs may negatively affect public health by providing selective pressure to increase antibiotic-resistant pathogens (*25*,*26*). Our study showed that, in addition to growing public health concerns about antibiotic resistance, some AGPs may facilitate the transmission of virulence factors in *E. coli* even at extremely low concentrations. Whereas the effect of monensin and tylosin on the propagation of Stx phages seemed to be marginal, chlortetracycline and oxytetracycline significantly induced the propagation of Stx phages (Figures 1, 2, 4) and mediated the transfer of Stx phages in *E. coli* (Figure 3).

**Figure 6:**
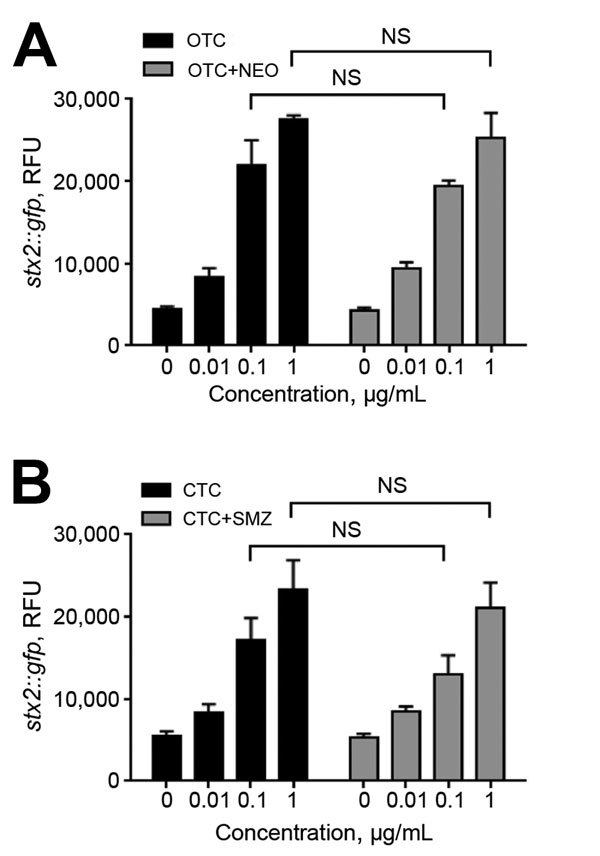
Induction of *stx2* expression by treatment of oxytetracycline (OTC) and chlortetracycline (CTC) in combination with other antibiotics. *Escherichia coli* O157 harboring *P_stx2_*::*gfp* was exposed to the following antibiotic combinations that are used in cattle for weight gain and feed efficiency: A) OTC/neomycin (NEO), and B) CTC/sulfamethazine (SMZ). The antibiotic combinations were prepared by mixing the indicated concentrations of each antibiotic. The results show means and SDs of a single representative experiment with triplicate samples. The experiment was repeated 3 times, and similar results were observed in all experiments. Statistical analysis was performed by using a Student *t*-test and GraphPad Prism 6 (http://www.graphpad.com/). NS, not significant; RFU, relative fluorescence units.

Tetracyclines are widely used in agriculture, accounting for 44% and 37% of marketed agricultural antibiotics in the United States (*27*) and the European Union (*28*), respectively. Compared with monensin and tylosin, oxytetracycline and chlortetracycline most significantly affected the transmission of Stx phages, even at concentrations as low as 0.01 μg/mL (Figure 3, panel B). This concentration is substantially lower than concentrations in the large intestines of cattle, which are 0.3 μg/mL after chlortetracycline feeding for growth promotion (70 mg/head/day throughout feedlot period) and 1.7 μg/mL after feeding for disease prevention (350 mg/head/day for 28 days) (*29*). A previous observational study reported that the percentage of detecting *stx*-positive commensal *E. coli* was increased in cattle from 48% to 80% by oxytetracycline injection and chlortetracycline addition to feed (*30*). Although they did not conclusively say that the increase in *stx*-positive animals is from oxytetracycline and chlortetracycline treatment in cattle, they suggested that antibiotic treatment may be the reason for the increased prevalence (*30*). Sometimes, chlortetracycline and oxytetracycline are mixed with other antibiotics, such as neomycin and sulfamethazine, to maintain weight gains and feed efficiency for cattle under stress conditions (*4*). We observed that tetracycline combinations with these antibiotics induced Stx phage propagation just as comparably as a single treatment of chlortetracycline or oxytetracycline alone (Figure 6), suggesting that oxytetracycline and chlortetracycline are the major bAGPs that induce propagation of Stx phages. Another concern about bAGPs would be associated with poor absorption of orally administered antibiotics in animal guts (*31*). Approximately 75% of dietary chlortetracycline is excreted in cattle manure without being digested (*32*), and chlortetracycline is the antimicrobial compound that is most frequently detected in cattle manure at levels as high as 20 mg/kg (*33*). Given the high residue concentrations in mature, unmetabolized tetracycline residues may also affect the dissemination of Stx phages in cattle manure.

Although use of chlortetracycline and oxytetracycline in cattle is not consistent (*4*), the levels of SOS response induction and Stx phage propagation by these 2 antibiotics were comparable (Figure 1). In addition to subtherapeutic use in feed, therapeutic concentrations of oxytetracycline and chlortetracycline are sometimes added to feed as metaphylaxis in feedlot cattle (*6*). We observed that chlortetracycline induced propagation of Stx phages more significantly at high concentrations than at low subtherapeutic concentrations (Figure 4). Surprisingly, propagation of Stx phages by 1 μg/mL oxytetracycline was comparable to that of 8 μg/mL chlortetracycline (Figure 4), suggesting that oxytetracycline is highly effective in phage induction. Possibly, therapeutic administration of chlortetracycline, oxytetracycline, and other antibiotics, particularly those inducing the SOS response and Stx phage propagation (e.g., fluoroquinolones) (*12*), would significantly affect the spread of Stx phages; however, its effect would be limited because therapeutic antibiotics are usually used to treat disease in individual animals.

Previous studies have reported that antibiotic treatment significantly increases the propagation of Stx phages (*24**, **34**–**36*). However, little attention has been paid to the effects of nonprescription bAGPs on the transmission of Stx phages in *E. coli*, although phages are a well-known vehicle for horizontal gene transfer and cattle are the primary reservoirs for *E. coli* O157:H7. Presumably, the underestimation of bAGPs might result from low concentrations of antibiotics in cattle feed. Nevertheless, in this study, we demonstrated that some bAGPs, particularly chlortetracycline and oxytetracycline, are implicated in the diversification of *stx*-positive O serotypes in *E. coli* by facilitating the horizontal transfer of Stx phages even at substantially low concentrations. Thus, use of these agents could lead to emergence of pathogenic *E. coli.*
